# Non-HLA Antibodies and Epitope Mismatches in Kidney Transplant Recipients With Histological Antibody-Mediated Rejection

**DOI:** 10.3389/fimmu.2021.703457

**Published:** 2021-07-06

**Authors:** Marta Crespo, Laura Llinàs-Mallol, Dolores Redondo-Pachón, Carrie Butler, Javier Gimeno, María José Pérez-Sáez, Carla Burballa, Anna Buxeda, Carlos Arias-Cabrales, Montserrat Folgueiras, Sara Sanz-Ureña, Nicole M. Valenzuela, Elaine F. Reed, Julio Pascual

**Affiliations:** ^1^ Department of Nephrology, Hospital del Mar, Barcelona, Spain; ^2^ Hospital del Mar Medical Research Institute (IMIM), Barcelona, Spain; ^3^ UCLA Immunogenetics Center, University of California Los Angeles, Los Angeles, CA, United States; ^4^ Department of Pathology and Laboratory Medicine, David Geffen School of Medicine, University of California Los Angeles, Los Angeles, CA, United States; ^5^ Department of Pathology, Hospital del Mar, Barcelona, Spain

**Keywords:** kidney transplantation, antibody-mediated rejection, HLA antibodies, non-HLA antibodies, HLA epitope mismatch, AT_1_R antibodies

## Abstract

**Background:**

Correlation between antibody-mediated rejection (ABMR) and circulating HLA donor-specific antibodies (HLA-DSA) is strong but imperfect in kidney transplant (KT) recipients, raising the possibility of undetected HLA-DSA or non-HLA antibodies contributing to ABMR. Detailed evaluation of the degree of HLA matching together with the identification of non-HLA antibodies in KT may help to decipher the antibody involved.

**Methods:**

We retrospectively assessed patients with transplant biopsies scored following Banff’15 classification. Pre- and post-transplant serum samples were checked for HLA and non-HLA antibodies [MICA-Ab, angiotensin-II type-1-receptor (AT_1_R)-Ab, endothelin-1 type-A-receptor (ETAR)-Ab and crossmatches with primary aortic endothelial cells (EC-XM)]. We also analyzed HLA epitope mismatches (HLA-EM) between donors and recipients to explore their role in ABMR histology (ABMR_h_) with and without HLA-DSA.

**Results:**

One-hundred eighteen patients with normal histology (n = 19), ABMR_h_ (n = 52) or IFTA (n = 47) were studied. ABMR_h_ patients were HLA-DSA_pos_ (n = 38, 73%) or HLA-DSA_neg_ (n = 14, 27%). Pre-transplant HLA-DSA and AT_1_R-Ab were more frequent in ABMR_h_ compared with IFTA and normal histology cases (p = 0.006 and 0.003), without differences in other non-HLA antibodies. Only three ABMR_h_DSA_neg_ cases showed non-HLA antibodies. ABMR_h_DSA_neg_ and ABMR_h_DSA_pos_ cases showed similar biopsy changes and graft-survival. Both total class II and DRB1 HLA-EM were associated with ABMR_h_DSA_pos_ but not with ABMR_h_DSA_neg_. Multivariate analysis showed that pre-transplant HLA-DSA (OR: 3.69 [1.31–10.37], p = 0.013) and AT_1_R-Ab (OR: 5.47 [1.78–16.76], p = 0.003) were independent predictors of ABMR_h_DSA_pos_.

**Conclusions:**

In conclusion, pre-transplant AT_1_R-Ab is frequently found in ABMR_h_DSA_pos_ patients. However, AT_1_R-Ab, MICA-Ab, ETAR-Ab or EC-XM^+^ are rarely found among ABMR_h_DSA_neg_ patients. Pre-transplant AT_1_R-Ab may act synergistically with preformed or *de novo* HLA-DSA to produce ABMR_h_DSA_pos_ but not ABMR_h_DSA_neg_. HLA epitope mismatch associates with ABMR_h_DSA_pos_ compared with ABMR_h_DSA_neg_, suggesting factors other than HLA are responsible for the damage.

## Introduction

Correlation between the detection of HLA donor-specific antibodies (HLA-DSA) and antibody-mediated rejection (ABMR) is strong but imperfect in kidney transplant (KT) recipients (1–7). Not all patients with pre- or post-transplant HLA-DSA have ABMR damage in their biopsies (8). Different groups have tried to identify characteristics of HLA-DSA that may predict ABMR (9–12). There is also an active search for other invasive or non-invasive biomarkers for ABMR diagnosis (13–15). In the other hand, some patients have biopsies with histological findings suggestive of ABMR (ABMR_h_) without circulating HLA-DSA (16), generating the concept of the existence of ABMR_h_DSA_pos_ and ABMR_h_DSA_neg_ cases. There is still limited literature describing the incidence of this type of ABMR without HLA-DSA, evaluating if these cases collectively show different clinical or histological characteristics or if non-HLA antibodies may explain the damage. Besides, controversial results in outcomes comparing ABMR_h_DSA_pos_ and ABMR_h_DSA_neg_ cases have been reported (17, 18).

Based on the hypothesis that other antibodies may play a lead role in the case of ABMR histological damage with or without HLA-DSA, some groups have evaluated non-HLA antibodies in KT recipients (19, 20). Although first reports connecting non-HLA antibodies and graft outcomes were published in 2005 (19, 21), evidence is still weak and debated. Antibodies against specific alloantigens such as MICA (MICA-Ab) or MICB, or against autoantigens like angiotensin II type 1 receptor (AT_1_R-Ab), endothelin-1 type A receptor (ETAR-Ab), perlecan, agrin or vimentin, among others, have been reviewed recently (22). Some groups focused into the analysis of pathogenic antibodies directed against endothelial cells—which express some of those but also other antigens—with endothelial cell crossmatches (23–25). The increased evidence that the prevalence of non-HLA antibodies in KT recipients is high (26), together with the heterogeneous post-KT clinical course of patients included in these studies (25) hamper the correct identification of deleterious non-HLA antibodies. On the other hand, HLA epitope mismatch (HLA-EM) assessment has gained interest as an added immune monitoring tool to provide a more precise evaluation of HLA matching (27–29). HLA-EM has been previously associated with the development of *de novo* HLA-DSA (30) and ABMR (31). The clinical relevance of HLA-EM analysis remains under discussion and its application is not generalized yet.

Here, we systematically explored pre- and post-KT serum samples for HLA and different types of non-HLA antibodies: MICA-Ab, AT_1_R-Ab and ETAR-Ab, and other non-HLA antibodies performing crossmatches with primary aortic endothelial cells (EC-XM). Additionally, we evaluated pre-KT HLA-EM load. We focused on KT patients with biopsies with Banff category 2 diagnosis and compared them with two other Banff diagnosis: category 1 or no abnormalities (normal), as a usual control group, and category 5 or interstitial fibrosis and tubular atrophy (IFTA), damage with not clear pathogenicity to evaluate the potential role of non-HLA antibodies in this case (32).

## Materials and Methods

### Study Population and Design

Prospective observational study performed in KT patients active at our transplant program in Hospital del Mar. A total of 234 consecutive clinical and surveillance renal biopsies were performed in ABO compatible KT after a negative CDC crossmatch (February 2011–June 2015). Ninety-two biopsies fulfilling Banff 2015 categories 3, 4 and 6 were excluded and 142 biopsies achieving categories 1, 2 or 5 were selected. From these 142 biopsies, we selected only one biopsy per patient according to these criteria: the first biopsy obtained after 3 months post-transplantation, unless a biopsy with category 2 diagnosis was available. Five biopsies were excluded due to unsuitable serum samples. Finally, 118 biopsies corresponding to 118 patients were included in the study (**Supplementary Figure 1**). Demographical and clinical data were collected as previously described (33), and follow-up was done until graft-loss, death, 96 months post biopsy or July/2020. The study was approved by the Parc de Salut Mar Ethical Research Board (2010/3904/I) and all patients signed informed consents. All clinical and research activities reported are consistent with the Declarations of Istanbul and Helsinki.

### Histological Scoring and Classification of the Biopsies

Biopsies were performed for indication or follow-up (including HLA-DSA detection without graft dysfunction). Processing was undertaken as previously described (33). All biopsies were scored by a pathologist following Banff 2015 classification and assigned to any of the six Banff categories (33). Category 2 included biopsies that met the first two Banff 2015–2019 criteria for ABMR histology, fulfilling the suspicious or full diagnosis of ABMR in Banff 2015 classification.

### Sera Collection and Detection of HLA and Non-HLA Antibodies

One-hundred one available pre-KT and 118 post-KT serum samples collected contemporaneously to biopsies were retrospectively analyzed. HLA antibody testing (HLA-A, B, C, DRB and DQB) was performed as previously described (34) using Luminex HLA Single Antigen Bead assays (LABScreen, One Lambda, Canoga Park, CA). Antibodies against MICA antigens (*001, *002, *004, *007, *009, *012, *017, *018, *019, *027) were determined using LABScreen assay by Luminex Technology, according to the manufacturer’s specifications (One Lambda, CA). MICA-Ab were considered positive if mean fluorescence intensity >1,000. MICA typing for donors and recipients was not available. AT_1_R-Ab and ETAR-Ab were measured using enzyme-linked immunosorbent-based assays (35) (One Lambda, CA), diluted 1:100, tested in duplicate and read on an Epoch Microplate Spectrophotometer (Bio-Tek, Winooski, VT). Samples with AT_1_R-Ab or ETAR ≥10 U/ml were considered positive based on previous studies and our receiver operating curve analysis.

### Endothelial Cell Crossmatches

Primary human aortic endothelial cells (ECs) were isolated from aortic rings of explanted donor hearts (36). EC were cultured in M199 medium supplemented with 20% (vol/vol) FBS, penicillin–streptomycin (100 U/ml and 100 ug/ml; Invitrogen Life Technologies), sodium pyruvate (1 mM), heparin (90 ug/ml; Sigma-Aldrich) and EC growth supplement (20 mcg/ml; Fisher Scientific). ECs from passages 7–8 were frozen and used in the EC-XM. Two different ECs (phenotyped as follows, donor CAR: HLA A2, A68, B60, B65; and donor Y126: HLA A1, A11, B35, B37) were employed avoiding for each KT recipient any HLA class I match with the kidney graft which could yield a reaction towards donor-specific HLA antigens. A total of 2 ×10^5^ ECs were incubated 30 min with 100 ul patient serum on ice. ECs were washed three times and incubated with 50 mc of 1:400 diluted FITC-AffiniPure F(ab’)_2_ Fragment Goat Anti-Human IgG Fc fragment (Jackson ImmunoResearch Laboratories) for 30 min on ice. After three washes, cell fluorescence was analyzed on a FACSCalibur flow cytometer using CellQuest software (BD Biosciences). Gates for forward and side scatter measurements were set on EC, and a minimum of 10,000 events was acquired. Positive EC-XM threshold was set at two standard deviations (50 Median Channel Shift) above the mean of negative control serum tests. EC-XM were only performed in 83 pre and 103 post-KT cases due to insufficient sample.

### HLA Epitope Mismatch Characterization

HLAMatchmaker software according to the July 2020 update (ABC and DRDQDP Eplet Matching Program V3.1, http://www.epitopes.net) was used to define potential HLA-EM between donors and recipients (37). High-resolution typing for all donors and recipients was performed or inferred using the HaploStats tool (www.haplostats.org) selecting the most likely high-resolution typing for HLA-A, B, C, DR and DQ according to three-five highest haplotype frequencies in the population of each one (Caucasian, African American, Asian or Hispanic).

### Statistics

Data are presented as mean ( ± standard deviation), median, interquartile range, or number (percentage) based on data distribution. Comparisons between clinical variables were carried out using Student’s T test for parametric continuous variables and U Mann–Whitney or Kruskal–Wallis test for non-parametric data. Chi-squared or Fisher’s exact tests were used to test categorical variables. Survival analyses were performed using the Kaplan–Meier method using the log-rank test. Logistic regression analysis was used to estimate the odds ratio (OR) for ABMR_h_DSA_pos_ development. All variables with a p-value <0.10 in the univariate analysis were included in the multivariate analysis. Statistical analysis was performed using SPSS v.27.0 (IBM Corp., Armonk, NY, USA) and p-values <0.05 were considered statistically significant.

## Results

### Clinical Characteristics and Graft Survival

The selected 118 patients were grouped according to Banff diagnostic categories: category 1 or normal biopsy (n = 19), category 2 or ABMR histology (ABMR_h_, n = 52) and category 5 or IFTA (n = 47). Thirty patients (25.4%) lost their grafts and 13 died with a functioning graft (11%) during the study period. Death-censored graft survival 68 months after the biopsy [IQR 48–80] was worse in ABMR_h_ cases than in those with IFTA or normal biopsies (**Supplementary Figure 2**). Baseline characteristics showed that normal histology patients were more frequently males, whereas ABMR_h_ patients received grafts from younger donors and were more frequently retransplanted. ABMR_h_ biopsies were less frequently surveillance biopsies and were performed later post-KT. At biopsy time, ABMR_h_ patients had worse glomerular filtration rate (GFR) and higher proteinuria. Finally, IFTA patients were more frequently receiving calcineurin inhibitors and less on mTOR inhibitors (**Table 1**).

**Table 1:** Demographics and clinical characteristics of all included patients.

	Normal (n = 19)	ABMR_h_ (n = 52)	IFTA (n = 47)	p-value
Recipient age (years) [mean (SD)]	47.9 (12.9)	47.4 (15.2)	53.1 (14.9)	0.14
Recipient gender (female) (n, %)	3 (15.8)	27 (51.9)	20 (42.6)	**0.024**
Recipient race (caucasian) (n, %)	15 (78.9)	46 (88.5)	43 (91.5)	0.38
Type of donor (deceased) (n, %)	15 (78.9)	46 (88.5)	45 (95.7)	0.11
Donor age (years) [mean (SD)]	50.0 (13.4)	45.8 (17.5)	54.4 (16.2)	**0.039**
Underlying renal disease				
− Glomerular disease (n, %)	2 (10.5)	11 (21.2)	10 (21.3)	
− SLE and other autoimmune disease (n, %)	0 (0)	2 (3.8)	2 (4.3)	0.33
− Diabetes (n, %)	1 (5.3)	1 (1.9)	6 (12.8)	
− Other (n, %)	16 (84.2)	38 (73.1)	29 (61.7)	
Retransplantation (n, %)	2 (10.5)	16 (30.8)	5 (10.6)	**0.028**
Peak CDC cPRA (%) [mean (SD)]	3.2 (5.8)	10.6 (23.1)	6.4 (16.5)	0.29
Pretransplant HLA antibodies (SAB) (yes) (n, %)*	15 (78.9)	28 (71.8)	31 (72.1)	0.82
HLA mismatch Class I (A/B) [mean (SD)]	3.1 (0.9)	2.8 (1.0)	2.9 (1.3)	0.59
HLA mismatch Class I (C) [mean (SD)]	1.5 (0.7)	1.3 (0.7)	1.3 (0.7)	0.56
HLA mismatch Class II (DR) [mean (SD)]	1.3 (0.8)	1.2 (0.6)	1.2 (0.7)	0.65
HLA mismatch Class II (DQ) [mean (SD)]	0.7 (0.7)	0.9 (0.7)	0.8 (0.6)	0.82
Antilymphocyte induction (n, %)	0 (0)	12 (23.1)	9 (19.1)	0.10
Delayed graft function (n, %)	3 (15.8)	19 (36.5)	14 (29.8)	0.24
Acute cellular rejection < 3 months after KT (n, %)	2 (10.5)	11 (21.2)	3 (6.4)	0.15
**Clinical characteristics and graft function at biopsy**				
Surveillance biopsy (n, %)	13 (68.4)	7 (13.5)	25 (53.2)	**<0.001**
Biopsy time after KT (months) [median (IQR)]	13 [10–23]	45 [14–120]	13 [11–35]	**<0.001**
Time biopsy to serum (days) [median (IQR)]	0 [−56,+34]	0 [−1,+53]	−0.5 [−20,+34]	0.40
Serum creatinine (mg/dl) [mean (SD)]	1.42 (0.5)	1.92 (0.9)	1.86 (1.4)	0.23
Estimated GFR (ml/min) [mean (SD)]	65.5 (30.7)	45.1 (23.7)	50 (22.2)	**0.009**
Urine protein/creatinine ratio (mg/g) [median (IQR)]	135.6 [114–295]	549 [180–1181]	199 [133–375]	**<0.001**
**Immunosuppressive treatment at biopsy**				
Prednisone (n, %)	17 (89.5)	39 (75)	42 (89.4)	0.14
Calcineurin inhibitors (n, %)	15 (78.9)	39 (75)	44 (93.6)	**0.030**
Mycophenolic acid (n, %)	17 (89.5)	43 (82.7)	38 (80.9)	0.76
mTOR inhibitors (n, %)	5 (26.3)	17 (32.7)	5 (10.6)	**0.027**
**Follow-up**				
Graft loss (n, %)	2 (10.5)	27 (51.9)	14 (29.8)	**0.003**
Death-censored graft loss (n, %)	2 (10.5)	21 (40.4)	7 (14.9)	**0.005**
Time after biopsy (months) [median (IQR)]	74 [67–83]	59 [23–81]	68 [62–77]	**0.044**

ABMR_h_, antibody-mediated rejection histology; CDC, complement-dependent cytotoxicity; GFR, glomerular filtration rate; IFTA, interstitial fibrosis and tubular atrophy; IQR, interquartile range; KT, kidney transplantation; PRA, panel-reactive antibody; SAB, Single Antigen Bead assays; SD, standard deviation; SLE, systemic lupus erythematosus. *From 101 available samples pre-transplantation. The bold values represent those p-values that are statistically significant.

### Pretransplant HLA-DSA and Non-HLA Antibodies

#### Pre-Transplant HLA-DSA

**Figure 1:**
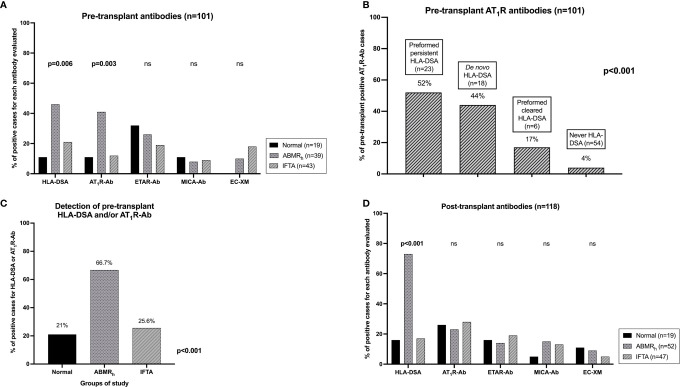
Pre-transplant HLA and non-HLA antibodies. **(A)** HLA, AT_1_R-Ab, ETAR-Ab, MICA-Ab and EC-XM before transplantation in the three groups of study. **(B)** Pre-transplant AT_1_R-Ab positive and negative patients with preformed persistent HLA-DSA, preformed cleared HLA-DSA, *de novo* HLA-DSA or without HLA-DSA at any time. **(C)** Detection of pre-transplant HLA-DSA and/or AT_1_R-Ab in the three study groups. **(D)** Post-transplant HLA and non-HLA antibodies. HLA, AT_1_R-Ab, ETAR-Ab, MICA-Ab and EC-XM after transplantation in the three groups of study. ABMR_h_, antibody-mediated rejection histology; AT_1_R-Ab, antibodies against angiotensin II type 1 receptor; EC-XM, crossmatch with primary aortic endothelial cells; ETAR-Ab, antibodies against endothelin-1 type A receptor; IFTA, interstitial fibrosis and tubular atrophy; KT, kidney transplantation; MICA-Ab, antibodies against major histocompatibility complex class I related chain A. ns, non-significant.

Pre-transplant serum samples were available for 101 patients (19 normal histology, 39 ABMR_h_ and 43 IFTA). We found pre-transplant HLA-DSA in 18 ABMR_h_ (46.2%), 9 IFTA (20.9%) and in two normal histology cases (10.5%) (p = 0.006) (**Figure 1A**). In ABMR_h_ pre-transplant HLA-DSA were more frequently class I&II combined (38.9%, p = 0.087) and less isolated class I (27.8%).

#### Pre-Transplant AT_1_R-Ab

Pre-transplant AT_1_R-Ab strongly associated with ABMR_h_ diagnosis (16/39 ABMR_h_ (41%) *vs.* 2/19 normal histology (10.5%) and 5/43 IFTA (11.6%), p = 0.003) (**Figure 1A**). All 16 ABMR_h_ patients with pre-transplant AT_1_R-Ab developed ABMR_h_DSA_pos_, whereas no ABMR_h_DSA_neg_ patient showed pre-transplant AT_1_R-Ab (p = 0.029). Detection of pre-transplant AT_1_R-Ab correlated with both persistent preformed HLA-DSA (12/23, 52%) and *de novo* HLA-DSA detection (8/18, 44%), but not with preformed HLA-DSA which cleared after transplant (1/6, 17%) or no HLA-DSA (2/54, 4%, p <0.001) (**Figure 1B**). The median MFI of preformed HLA-DSA coexistent with AT_1_R-Ab was not significantly different than preformed HLA-DSA without AT_1_R-Ab (8898 *vs* 2874, p = 0.083).

#### Other Non-HLA Antibodies

Neither pre-transplant ETAR-Ab nor MICA-Ab associated with ABMR_h_. Pre-transplant ETAR-Ab and MICA-Ab were present similarly in normal histology, ABMR_h_ and IFTA cases (31.6, 25.6 and 18.6%, p = 0.51; 10.5, 7.7 and 9.3%, p = 1.00). Of 83 KT recipients tested with EC-XM, only 3/29 ABMR_h_ (10.3%) and 3/39 IF/TA cases (7.7%) had a pre-transplant positive EC-XM (**Figure 1A**).

#### Pre-Transplant Combination of HLA-DSA and Non-HLA Antibodies

Detection of pre-transplant HLA-DSA and/or AT_1_R-Ab were highly associated with ABMR_h_ compared with IFTA and normal biopsies (66.7 *vs.* 25.6 *vs.* 21%, p <0.001, **Figure 1C**). Nine ABMR_h_ cases presented with simultaneous HLA-DSA and AT_1_R-Ab (23.1%), 17 with either HLA-DSA or AT_1_R-Ab (43.6%) and the remaining 13 did not present any of these antibodies (33.3%).

### Post-Transplant HLA-DSA and Non-HLA Antibodies

#### Post-Transplant HLA-DSA

At the time of biopsy, HLA-DSA was detectable in 38/52 ABMR_h_ patients [73.1%, 17 preformed (44.7%) and 21 *de novo* (55.3%)]. Among them, 7.7% were class I, 53.8% class II and 11.5% combined class I&II. HLA-DSA were also detected in 17% IFTA and 15.8% normal histology cases (**Figure 1D**).

#### Post-Transplant AT_1_R-Ab

Post-transplant AT_1_R-Ab showed no association with ABMR_h_ (23.1% in ABMR_h_ *vs.* 26.3% in normal histology and 27.7% in IFTA cases, p = 0.85, **Figure 1D**). Detection of post-transplant AT_1_R-Ab did not correlate with the detection of HLA-DSA [15/49 HLA-DSA_pos_ cases had AT_1_R-Ab at biopsy (30.6%) *vs.* 15/69 HLA-DSA_neg_ cases (21.7%), p = 0.28].

#### Other Non-HLA Antibodies

Neither post-transplant ETAR-Ab nor MICA-Ab was related with ABMR_h_. Post-transplant ETAR-Ab were found in 3/19 normal histology (15.8%), 7/52 ABMR_h_ (13.5%) and 9/47 IFTA cases (19.1%, p = 0.80). MICA-Ab were detectable in 1/19 normal histology (5.3%), 8/52 ABMR_h_ (15.4%) and 6/47 IFTA cases (12.8%, p = 0.62). Two normal histology (11.1%), four ABMR_h_ (9.3%) and two IFTA cases (4.8%) had a positive EC-XM (p = 0.70, **Figure 1D**).

### Patients With ABMR_h_ With and Without HLA-DSA

**Figure 2:**
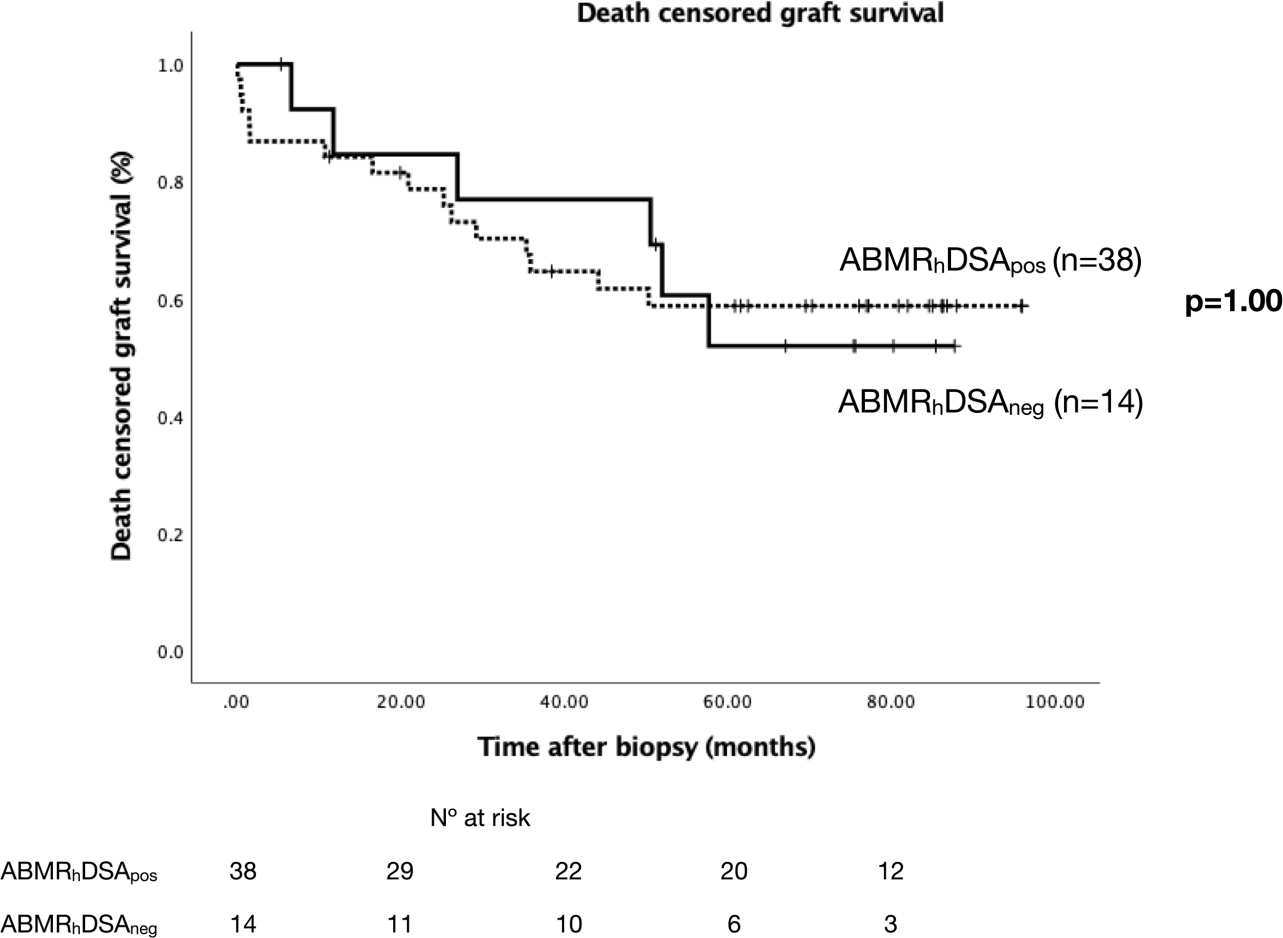
Death censored graft survival in ABMR_h_ patients with and without HLA-DSA. Kaplan–Meier survival curves representing death censored graft survival. ABMR_h_, antibody-mediated rejection histology; DSA, donor-specific antibodies.

From 52 patients with ABMR_h_ 14 (26.9%) had no peri-biopsy HLA-DSA. ABMR_h_DSA_pos_ cases were more frequently HLA sensitized, less well DR-matched with their donors and received more frequently a graft from a deceased donor than those ABMR_h_DSA_neg_. No differences were found in graft function or immunosuppression at biopsy (**Table 2**). Patients showed similar microvascular inflammation, but diffuse C4d was more frequent in ABMR_h_DSA_pos_ cases (27% *vs* 0%, p = 0.07, **Table 2**). Graft survival was similar between both groups (**Figure 2**). We assessed pre- and post-transplant non-HLA antibodies in ABMR_h_DSA_neg_ cases. Of 7 cases with pre-transplant sample, two had EC-XM^+^ but none showed MICA-Ab, AT_1_R-Ab or ETAR-Ab (**Table 3A**). After KT, one had coexistent MICA-Ab, AT_1_R-Ab and ETAR-Ab; one had MICA-Ab and a third one AT_1_R-Ab (**Supplementary Table 1A**). In 9/14 ABMR_h_DSA_neg_ patients (64.3%) we could not identify any of the non-HLA antibodies studied.

**Table 2:** Characteristics of patients with and without HLA-DSA.

	ABMR_h_DSA_pos_ (n = 38)	ABMR_h_DSA_neg_ (n = 14)	p-value
Recipient age (years) [mean (SD)]	47.8 (15.7)	46.4 (14.2)	0.76
Recipient gender (female) (n, %)	20 (52.6)	7 (50)	1.00
Recipient race (caucasian) (n, %)	34 (89.5)	12 (85.7)	0.46
Type of donor (deceased) (n, %)	36 (94.7)	10 (71.4)	**0.038**
Donor age (years) [mean (SD)]	45.5 (18.9)	46.9 (13.7)	0.80
Underlying renal disease			
− Glomerular disease (n, %)	5 (13.2)	6 (42.9)	0.10
− SLE and other autoimmune disease (n, %)	2 (5.3)	0 (0)	
− Diabetes (n, %)	1 (2.6)	0 (0)	
− Other (n, %)	30 (78.9)	8 (57.1)	
Retransplantation (n, %)	14 (36.8)	2 (14.3)	0.18
Peak CDC cPRA (%) [mean (SD)]	14.2 (26.2)	0.6 (2.4)	**0.003**
Pretransplant HLA antibodies (SAB) (yes) (n, %)*	25 (78.1)	3 (42.9)	0.08
HLA mismatch Class I (A/B) [mean (SD)]	2.8 (1.0)	2.6 (1.0)	0.52
HLA mismatch Class I (C) [mean (SD)]	1.3 (0.7)	1.1 (0.8)	0.25
HLA mismatch Class II (DR) [mean (SD)]	1.4 (0.5)	0.7 (0.6)	**<0.001**
HLA mismatch Class II (DQ) [mean (SD)]	0.9 (0.7)	0.7 (0.7)	0.41
Antilymphocyte induction (n, %)	9 (23.7)	3 (21.4)	0.28
Delayed graft function (n, %)	16 (42.1)	3 (21.4)	0.21
Acute cellular rejection <3 months after KT (n, %)	5 (13.2)	6 (42.9)	0.08
**Clinical characteristics and graft function at biopsy**			
Surveillance biopsy (n, %)	18 (47.4)	4 (28.6)	0.34
Biopsy time after KT (months) [median (IQR)]	44 [14–99]	74 [15–220]	0.22
Time biopsy to serum (days) [mean (SD)]	30 (78)	20 (61)	0.66
Serum creatinine (mg/dl) [mean (SD)]	2.01 (1.0)	1.70 (0.6)	0.30
Estimated GFR (ml/min) [mean (SD)]	44.8 (25.5)	45.8 (19.1)	0.89
Urine protein/creatinine ratio (mg/g) [median (IQR)]	413 [170–1189]	695 [406–1,174]	0.27
**Immunosuppressive treatment at biopsy**			
Prednisone (n, %)	30 (78.9)	9 (64.3)	0.30
Calcineurin inhibitors (n, %)	27 (71.1)	12 (85.7)	0.47
Mycophenolic acid (n, %)	32 (84.2)	11 (78.6)	0.69
mTOR inhibitors (n, %)	14 (36.8)	3 (21.4)	0.34
**Follow-up**			
Graft loss (n, %)	19 (50)	8 (57.1)	0.76
Death-censored graft loss (n, %)	15 (39.5)	6 (42.9)	1.00
Time after biopsy (months) [median (IQR)]	61 [21–85]	55 [27–76]	0.87
**Histological features of ABMR_h_**			
Percentage of glomerulosclerosis [mean (SD)]	18.4% (17.5)	18.8% (18.4)	0.95
Glomerulitis (g ≥1) (yes, %)	30 (78.9)	12 (85.7)	0.71
g0	8 (21.1)	2 (14.3)	
g1	16 (42.1)	4 (28.6)	0.58
g2	10 (26.3)	5 (35.7)	
g3	4 (10.5)	3 (21.4)	
Peritubular capilaritis (ptc ≥1) (yes, %)	31 (81.6)	9 (64.3)	0.27
ptc0	7 (18.4)	5 (35.7)	
ptc1	21 (55.3)	5 (35.7)	0.18
ptc2	10 (26.3)	3 (21.5)	
ptc3	0 (0)	1 (7.1)	
Microvascular inflammation (g + ptc ≥2) (yes, %)	31 (81.6)	12 (85.7)	1.00
C4d positivity (yes, %)	17 (44.7)	6 (42.9)	1.00
C4d0	20 (54.1)	8 (57.1)	
C4d1	4 (10.8)	2 (14.3)	0.07
C4d2	3 (8.1)	4 (28.6)	
C4d3	10 (27.0)	0 (0)	
Chronic transplant glomerulopathy (yes, %)^#^	20 (58.9)	9 (69.2)	0.74
EM CTG or PTCML (yes, %)^#^	28 (82.4)	9 (69.2)	0.43
Arteriolar hialinosis (ah ≥1) (yes, %)	18 (47.4)	6 (42.9)	0.76
Arterial intimal fibrosis (cv ≥1) (yes, %)^#^	18 (52.9)	6 (50)	1.00
Interstitial fibrosis (ci ≥1) (yes, %)	35 (92.1)	14 (100)	0.56
Tubular atrophy (ct ≥1) (yes, %)	32 (84.2)	14 (100)	0.17
Tubulitis (t ≥1) (yes, %)	8 (21.1)	0 (0)	0.09
Interstitial inflammation (i ≥1) (yes, %)	6 (15.8)	0 (0)	0.17
Intimal arteritis (v ≥1) (yes, %)^#^	1 (3.1)	0 (0)	1.00

ABMR_h_, antibody-mediated rejection histology; CDC, complement-dependent cytotoxicity; CTG, chronic transplant glomerulopathy; EM, electron microscopy; GFR, glomerular filtration rate; IFTA, interstitial fibrosis and tubular atrophy; IQR, interquartile range; KT, kidney transplantation; PRA, panel-reactive antibody; PTCML, peritubular capillary multilayering; SAB, Single Antigen Bead assays; SD, standard deviation; SLE, systemic lupus erythematosus. *From 101 available samples pre-transplantation. ^#^From 46/47 biopsies (34 ABMR_h_DSA_pos_, 12/13 ABMR_h_DSA_neg_). The bold values represent those p-values that are statistically significant.

**Table 3A:** Comparison of pre-transplant non-HLA antibodies between ABMR_h_DSA_pos_ and ABMR_h_DSA_neg_ cases.

	ABMR_h_DSA_pos_ (n = 38)*	ABMR_h_DSA_neg_ (n = 14)*	p-value
Pre-transplant AT_1_R-Ab (yes, %)	16 (50)	0 (0)	**0.029**
Pre-transplant ETAR-Ab (yes, %)	10 (31.3)	0 (0)	0.16
Pre-transplant MICA-Ab (yes, %)	3 (9.4)	0 (0)	1.00
Pre-transplant EC-XM (positive, %)^#^	1 (4.5)	2 (28.6)	0.14

*From 32 ABMR_h_DSA_pos_ and 7 ABMR_h_DSA_neg_ cases with pre-transplant available samples. ^#^From 22 ABMR_h_DSA_pos_ and 7 ABMR_h_DSA_neg_ cases. The bold values represent those p-values that are statistically significant.

### HLA Epitope Mismatch Characterization

**Figure 3:**
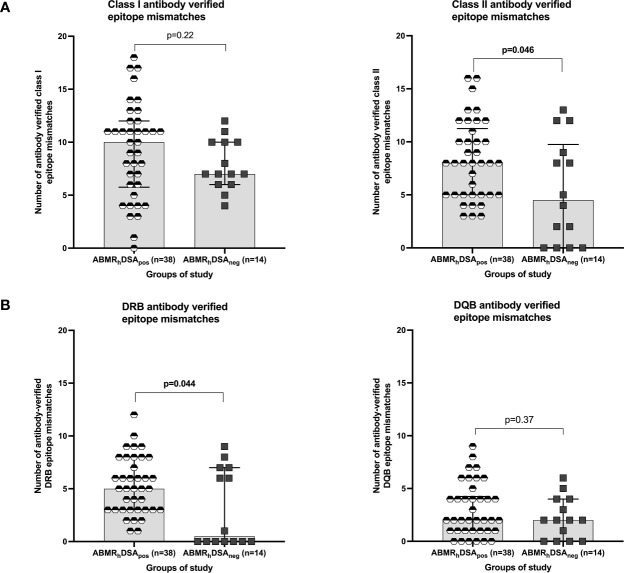
HLA epitope mismatch analysis in ABMR_h_DSA_pos_ and ABMR_h_DSA_neg_ cases. **(A)** Number of antibody-verified class I and class II epitope mismatches and **(B)** Number of antibody-verified DRB and DQB epitope mismatches in ABMR_h_DSA_pos_ (black and white hexagons) and ABMR_h_DSA_neg_ (black squares) cases. All plots show median and interquartile range (IQR).

The median number of class I and class II HLA-EM in our cohort were 16 (0–36) and 18 (0–46) respectively. Among them, 10 class I and 7 class II HLA-EM were antibody-verified (HLA-EM^ver^). We observed similar class I and class II HLA-EM^ver^ in all three groups of study (data not shown). We compared the load of HLA-EM^ver^ between ABMR_h_DSA_pos_ and ABMR_h_DSA_neg_ patients, finding similar class I but significantly higher class II and DRB HLA-EM^ver^ in ABMR_h_DSA_pos_ cases (8 *vs* 4.5, p = 0.046; 5 *vs.* 0.5, p = 0.044, **Figure 3**). We compared HLA-EM and HLA antigen mismatch (HLA-AM) for *de novo* DSA (dnDSA) development prediction. Neither class I HLA-EM^ver^ nor HLA-AM were useful tools for class I dnDSA prediction. Class II HLA-EM^ver^ were significantly associated with class II dnDSA (8 *vs.* 7, p = 0.031), but not class II HLA-AM (p = 0.26). The extent of DRB HLA-EM^ver^ associated with DRB dnDSA (6 *vs.* 4, p = 0.024), and the rate of DQB HLA-EM^ver^ showed a weak association with DQB dnDSA (4 *vs.* 2, p = 0.077). Neither DRB nor DQB HLA-AM predicted DRB or DQB dnDSA (p = 0.27, p = 0.21).

### Risk Factors for Post-Transplant ABMR_h_DSA_pos_ Development

ABMR_h_DSA_pos_ patients showed higher rates of pre-transplant HLA-DSA and AT_1_R-Ab (p <0.001, **Table 3B**), but regarding post-transplant antibodies, only HLA-DSA was associated with ABMR_h_DSA_pos_ (p <0.001, **Supplementary Table 1B**). In order to assess the role of each factor, we adjusted a multivariate model which showed that both pre-transplant HLA-DSA (OR: 3.69 [1.31–10.37], p = 0.013) and AT_1_R-Ab (OR: 5.47 [1.78–16.76], p = 0.003) were independent ABMR_h_DSA_pos_ predictors. DRB HLA-EM^ver^ also showed a weak association with ABMR_h_DSA_pos_ (p = 0.071, **Table 4**).

**Table 3B:** Pre-transplant HLA and non-HLA antibodies: comparison between ABMR_h_DSA_pos_ and non-ABMR_h_DSA_pos_ cases (normal histology, IFTA and ABMR_h_DSA_neg_ cases).

	ABMR_h_DSA_pos_ (n = 38)*	No ABMR_h_DSA_pos_ (n = 80)*	p-value
Pre-transplant HLA-DSA (yes, %)	17 (53.1)	12 (17.4)	**<0.001**
Pre-transplant AT_1_R-Ab (yes, %)	16 (50)	7 (10.1)	**<0.001**
Pre-transplant ETAR-Ab (yes, %)	10 (31.2)	14 (20.3)	0.23
Pre-transplant MICA-Ab (yes, %)	3 (9.4)	6 (8.7)	1.00
Pre-transplant EC-XM (positive, %)^$^	1 (4.5)	5 (8.2)	1.00

*32 ABMR_h_DSA_pos_ cases and 69 non-ABMR_h_DSA_pos_ cases with pre-transplant available samples. ^$^22 ABMR_h_DSA_pos_ and 61 non-ABMR_h_DSA_pos_ cases.

**Table 4:** Logistic regression analysis of ABMR_h_DSA_pos_ risk factors.

	Univariate		Multivariate	
Risk factor	OR (95% CI)	p-value	OR (95% CI)	p-value
Pre-transplant HLA-DSA	5.38 (2.12–13.68)	**<0.001**	3.69 (1.31-10.37)	**0.013**
Pre-transplant AT_1_R-Ab	8.86 (3.12–25.17)	**<0.001**	5.47 (1.78-16.76)	**0.003**
Pre-transplant ETAR-Ab	1.79 (0.69–4.62)	0.23		
Pre-transplant MICA-Ab	1.09 (0.25–4.65)	0.91		
Pre-transplant positive EC-XM	0.53 (0.06–4.84)	0.58		
Class I HLA-EM^ver^	0.99 (0.90–1.08)	0.79		
DRB HLA-EM^ver^	1.21 (1.04–1.40)	**0.011**	1.18 (0.99-1.41)	0.071
DQB HLA-EM^ver^	1.10 (0.94–1.29)	0.23		

AT_1_R-Ab, antibodies against angiotensin II type 1 receptor; EC-XM, crossmatch with primary aortic endothelial cells; ETAR-Ab, antibodies against endothelin-1 type A receptor; HLA-DSA, HLA donor-specific antibodies; HLA-EM^ver^, antibody-verified HLA epitope mismatches; MICA-Ab, antibodies against major histocompatibility complex class I related chain A. The bold values represent those p-values that are statistically significant.

## Discussion

We report here that ABMR damage in KT recipients occurs in a significant proportion of cases without the detection of HLA-DSA at biopsy. We have evaluated the role of non-HLA antibodies, such as AT_1_R-Ab, ETAR-Ab, MICA-Ab or anti-EC antibodies detected with crossmatches and found they could not explain ABMR_h_DSA_neg_. Our results suggest a synergistic interaction between pre-transplant AT_1_R-Ab and HLA-DSA to produce ABMR_h_DSA_pos_ or facilitate *de novo* appearance of HLA-DSA, but not to induce ABMR_h_DSA_neg_. Interestingly, it appears more strongly associated with ABMR_h_ than incompatibility evaluated through HLA-EM analysis.

The relationship between ABMR_h_ and HLA-DSA has been described in KT recipients for over 20 years (1, 2). However, there is increased evidence that ABMR compatible histological lesions may be present in the graft without detectable circulating HLA-DSA (18, 38). Up to 27% of our ABMR_h_ patients did not show circulating HLA-DSA at the time of biopsy. This could be attributed to the inability of current techniques to detect these HLA antibodies or due to the participation of a different set of antibodies in graft damage. ABMR_h_DSA_neg_ patients presented significantly lower class II and DRB HLA-EM compared with ABMR_h_DSA_pos_ cases. This finding strengthens the hypothesis of the participation of other mechanisms of damage in these cases rather than non-detected HLA-DSA. However, neither AT_1_R-Ab, ETAR-Ab, MICA-Ab nor antibodies identified with EC-XM before or after KT were able to explain the ABMR_h_DSA_neg_ cases in our study. We describe here that ABMR_h_ patients without HLA-DSA showed similar graft function, immunosuppressive treatment, histological features at biopsy and graft survival at the end of follow-up compared with ABMR_h_DSA_pos_ cases. Like us, Sablik et al. (17) reported a similar histological phenotype in ABMR_h_DSA_pos_ and ABMR_h_DSA_neg_ patients, but a larger study by Senev et al. (18) found that ABMR_h_DSA_pos_ biopsies were more frequently C4d positive compared with ABMR_h_DSA_neg_ cases, as the unique histological difference between the groups. In our series, although C4d positivity was similar between both groups, C4d intensity was higher in the ABMR_h_DSA_pos_ group. In our cohort, graft survival was similar between both groups, in agreement with results reported by Sablik et al. (17) but in contrast with the study from Senev et al. (18), which included mostly active ABMR cases without chronicity, unlike our cohort.

KT recipients may produce immune responses through indirect recognition against foreign proteins or even against own proteins expressed by the donor graft acting as autoantigens due to different factors that induce graft damage during the transplant process. These antibodies may then react against polymorphic alloantigens, like HLA related MICA or MICB, or against autoantigens like AT_1_R, ETAR, agrin, vimentin, perlecan, K-tubulin, etc. (39–41) which may be prevalent in KT recipients. Some of these autoantibodies and new ones recently validated (42) have not been evaluated in our cohort yet. They might explain some ABMR_h_DSA_neg_ cases. Some groups have evaluated the relationship between antibodies directed against ECs—the barrier between donor and recipient—and graft survival (43), and exploratory studies have employed array techniques in limited series with antibodies against ECs validating potential target proteins with ELISA (44, 45). Jackson and col. were able to identify four antigenic targets expressed on ECs in nine patients with ABMR_h_DSA_neg_ (44). They found that antibodies against these proteins in pre-transplant sera predicted ABMR_h_DSA_pos_. In our cohort, of seven ABMR_h_DSA_neg_ cases with pre-transplant samples, two had a positive EC-XM^+^, but none showed MICA-Ab, AT_1_R-Ab or ETAR-Ab. In line with our results, a recent report from Delville et al. (23) found that only 26% of patients with early acute ABMR_h_DSA_neg_ had pre-transplant AT_1_R-Ab using our same threshold of 10 UI/ml. Moreover, MICA-Ab were only detected in two of these ABMR_h_DSA_neg_ cases. However, these cases had preformed IgG antibodies against constitutively expressed antigens of microvascular glomerular cells (23). Of note, our two cases with pre-transplant EC-XM^+^ developed ABMR_h_ within the first 12 months of KT, while the other twelve developed ABMR_h_ later on. Unlike Lefaucheur et al. (46), despite employing the same threshold for AT_1_R antibodies, the presence of these antibodies in our ABMR_h_DSA_neg_ cohort is negligible. Nevertheless, our overall prevalence of 25% in post-transplant AT_1_R-Ab is not different from theirs. Unfortunately, these authors do not analyze the relation between pre-transplant AT_1_R-Ab and ABMR.

We report here a strong and independent association between pre-transplant AT_1_R-Ab and ABMR_h_DSA_pos_ development. AT_1_R can be found in several cell types such as vascular endothelial cells and binds to angiotensin II (39, 47). First report linking AT_1_R-Ab and kidney allograft rejection suggested a potential relationship between AT_1_R agonistic antibodies and vascular injury (19, 39). Subsequently, pre- or post-transplant AT_1_R-Ab detection have been linked to both rejection and allograft failure (19, 48). Philogene et al. (24) described higher post-transplant AT_1_R-Ab levels in patients with ABMR compared with patients with cellular rejection or those without rejection, however, they provided no data regarding pre-transplant AT_1_R-Ab. In another report (49), pre- and post-transplant AT_1_R-Ab were strongly associated with biopsy-proven rejection, not specifically ABMR. Some reports suggest that non-HLA and HLA-DSA antibodies may function in synergy (24, 49). Taniguchi et al. (49) reported lower graft survival mainly in the presence of *de novo* AT_1_R-Ab and HLA-DSA at biopsy with lesions compared with those cases with HLA-DSA alone. Here we show a strong association of pre-transplant AT_1_R-Ab with post-transplant HLA-DSA, either persistent preformed or *de novo*, and with ABMR_h_DSA_pos_ development. This association may be of utmost importance for KT outcomes. We previously reported the strong association among persistent preformed HLA-DSA and lower ABMR free survival, only surpassed by the development of *de novo* HLA-DSA (34). Moreover, here we show that all 16 ABMR_h_ patients with pre-transplant AT_1_R-Ab had HLA-DSA at biopsy, nine of them maintained the preformed HLA-DSA and seven developed *de novo* HLA-DSA. We found no association between pre-transplant AT_1_R-Ab and graft survival, in line with other reports (26, 49). In our multivariate analysis, pre-transplant HLA-DSA and AT_1_R-Ab were independent predictors for ABMR_h_. Our study may not be powered enough to assess the relationship between AT_1_R-Ab and graft loss. Given the strong and already known association between ABMR and increased risk of kidney allograft loss (34, 50–52), our data supports that pre-transplant AT_1_R-Ab assessment should be carefully considered in KT candidates.

In the last years, HLA-EM analysis has been proposed as a better strategy to prevent HLA-DSA development than antigen matching (29). Here we confirm that class II and DRB dnDSA development may be predicted with HLA-EM, as previously reported (30), however, only a weak association was observed with DQB dnDSA, probably due to the limited number of cases included. Interestingly, neither class II, DRB or DQB HLA-AM were able to predict dnDSA. As mentioned, the detection of lower number of class II and DRB HLA-EM in ABMR_h_DSA_neg_ cases may contradict the idea of undetected HLA-DSA responsible for the damage. Class II and DRB HLA-EM associated with ABMR_h_DSA_pos_, although the existence of preformed HLA-DSA or AT_1_R-Ab are more potent predictors of ABMR_h_DSA_pos_ in our experience. In our study, ABMR_h_DSA_neg_ could not be explained by higher HLA-EM or by the non-HLA antibodies evaluated. Interestingly, an alternative mechanism to produce ABMR_h_ termed “the missing-self hypothesis” has been proposed. According to it, the inability of graft EC to provide HLA I-mediated inhibitory signals to recipient circulating NK cells may trigger NK cell activation, resulting in endothelial damage and chronic vascular rejection (53).

The main limitation of our study is the restricted number of ABMR_h_DSA_neg_ cases in the whole cohort. In order to further increase its number and the significance of the study, a multicenter trial is advisable. Besides, it is based on a mix of indication and surveillance biopsies which introduces heterogeneity in the timing and clinical picture of patients. Of note, EC-XM were performed with aortic cells which may not express the same proteins as a renal EC. Last, another limitation may be the use of inferred four-digit HLA typing for HLA-EM analysis. Despite careful estimation of second field HLA typing, we cannot rule out the possibility that some rare HLA genotypes are not correctly assigned, as recently suggested (54). However, ours is a large well characterized cohort of KT recipients, reflecting clinical practice, with thorough analysis of biopsies, including electron microscopy, crucial to detect some cases of ABMR_h_ and with systematical study of HLA-DSA and a known set of non-HLA antibodies.

In summary, although the majority of patients with HLA-DSA at the time of biopsy show ABMR_h_, almost 30% of ABMR_h_ patients did not show evidence of circulating HLA-DSA. These patients were more frequently HLA unsensitized pretransplant and less HLA matched but did not show other specific characteristics at transplantation or at biopsy. Neither AT_1_R-Ab, ETAR-Ab, MICA-Ab nor antibodies identified with EC-XM before or after KT were able to explain ABMR_h_DSA_neg_ cases. Importantly AT_1_R-Ab with or without HLA-DSA before KT clearly increased the risk of ABMR_h_DSA_pos_, suggesting it should be included in the pre-transplant immune assessment together with HLA-DSA.

## Data Availability Statement

The raw data supporting the conclusions of this article will be made available by the authors, without undue reservation.

## Ethics Statement

The studies involving human participants were reviewed and approved by the Parc de Salut Mar Ethical Research Board. The patients/participants provided their written informed consent to participate in this study.

## Author Contributions

MC designed the study, coordinated logistics, analyzed the results, and drafted the manuscript. LL-M analyzed the results and drafted the manuscript. DR-P analyzed the results and revised the manuscript. CBut coordinated lab procedures and revised the manuscript. JG contributed with the assessment of the graft biopsies. MP-S, CBur, AB, CA-C, and SS-U revised the manuscript. MF coordinated sample drawing and storage. NV supervised HLA and non-HLA antibody interpretation. ER and JP evaluated the design of the study and revised the manuscript. All authors contributed to the article and approved the submitted version.

## Funding

This study was performed with funding from projects PI13/00598, PI16/00617, and PI20/00090 (Spanish Ministry of Health ISCIII FIS-FEDER); RD16/0009/0013 (ISCIII FEDER REDinREN) and 201822-10 (Fundació la Marató de TV3). MC received grants of the Sociedad Española de Nefrología and Parc de Salut Mar for a research stay at UCLA Immunogenetics Center (Los Angeles, USA). ER is supported by National Institute of Allergy and Infectious Diseases Grants RO1 AI135201. One Lambda provided reagents but had no role in the design of the study or the analyses and writing of the manuscript.

## Conflict of Interest

The authors declare that the research was conducted in the absence of any commercial or financial relationships that could be construed as a potential conflict of interest.

## Abbreviations

ABMR, antibody-mediated rejection; ABMR_h_, antibody-mediated rejection histology; ABMR_h_DSA_pos_, antibody-mediated rejection histology with HLA-DSA; ABMR_h_DSA_neg_, antibody-mediated rejection histology without HLA-DSA; AT_1_R-Ab, antibodies against angiotensin II type 1 receptor; CDC, complement-dependent cytotoxicity; CTG, chronic transplant glomerulopathy; ECs, primary human aortic endothelial cells; EC-XM, crossmatch with primary human aortic endothelial cells; EM, electron microscopy; ETAR-Ab, antibodies against endothelin-1 type A receptor; GFR, glomerular filtration rate; HLA-DSA, HLA donor-specific antibodies; HLA-AM, HLA antigen mismatches; HLA-EM, HLA epitope mismatches; IFTA, interstitial fibrosis and tubular atrophy; IQR, interquartile range; KT, kidney transplant; MICA-Ab, antibodies against major histocompatibility complex class I related chain A; PRA, panel-reactive antibody; PTCML, peritubular capillary multilayering; SAB, Single Antigen Bead assays; SD, standard deviation.

## References

[1] Crespo M Pascual M Tolkoff-Rubin N Mauiyyedi S Bernard Collins A Fitzpatrick D. Acute Humoral Rejection in Renal Allograft Recipients: I. Incidence, Serology and Clinical Characteristics. Transplantation (2001) 71(5):652–8. 10.1097/00007890-200103150-00013 11292296

[2] Terasaki PI Ozawa M. Predicting Kidney Graft Failure by HLA Antibodies: A Prospective Trial. Am J Transplant (2004) 4:438–43. 10.1111/j.1600-6143.2004.00360.x 14961999

[3] Djamali A Kaufman DB Ellis TM Zhong W Matas A Samaniego M. Diagnosis and Management of Antibody-Mediated Rejection: Current Status and Novel Approaches. Am J Transplant (2014) 14:255–71. 10.1111/ajt.12589 PMC4285166 24401076

[4] Lefaucheur C Viglietti D Mangiola M Loupy A Zeevi A. From Humoral Theory to Performant Risk Stratification in Kidney Transplantation. J Immunol Res (2017) 2017:10–8. 10.1155/2017/5201098 PMC5241462 28133619

[5] Viglietti D Loupy A Vernerey D Bentlejewski C Gosset C Aubert O. Value of Donor-Specific Anti-HLA Antibody Monitoring and Characterization for Risk Stratification of Kidney Allograft Loss. J Am Soc Nephrol (2017) 28:702–15. 10.1681/ASN.2016030368 PMC5280026 27493255

[6] Wiebe C Gibson IW Blydt-Hansen TD Karpinski M Ho J Storsley LJ. Evolution and Clinical Pathologic Correlations of De Novo Donor-Specific HLA Antibody Post Kidney Transplant. Am J Transplant (2012) 12:1157–67. 10.1111/j.1600-6143.2012.04013.x 22429309

[7] Eskandary F Bond G Kozakowski N Regele H Marinova L Wahrmann M. Diagnostic Contribution of Donor-Specific Antibody Characteristics to Uncover Late Silent Antibody-Mediated Rejection-Results of a Cross-Sectional Screening Study. Transplantation (2017) 101(3):631–41. 10.1097/TP.0000000000001195 27120452

[8] Schinstock CA Cosio F Cheungpasitporn W Dadhania DM Everly MJ Samaniego-Picota MD. The Value of Protocol Biopsies to Identify Patients With De Novo Donor-Specific Antibody at High Risk for Allograft Loss. Am J Transplant (2017) 17(6):1574–84. 10.1111/ajt.14161 PMC5445019 27977905

[9] Crespo M Torio A Mas V Redondo D Perez-Saez MJ Mir M. Clinical Relevance of Pretransplant Anti-HLA Donor-Specific Antibodies: Does C1q-Fixation Matter? Transpl Immunol (2013) 29:28–33. 10.1016/j.trim.2013.07.002 23907088

[10] Loupy A Lefaucheur C Vernerey D Prugger C Duong van Huyen JP Mooney N. Complement-Binding Anti-HLA Antibodies and Kidney-Allograft Survival. N Engl J Med (2013) 369(13):1215–26. 10.1056/NEJMoa1302506 24066742

[11] Sicard A Ducreux S Rabeyrin M Couzi L McGregor B Badet L. Detection of C3d-Binding Donor-Specific Anti-HLA Antibodies at Diagnosis of Humoral Rejection Predicts Renal Graft Loss. J Am Soc Nephrol (2015) 26:457–67. 10.1681/ASN.2013101144 PMC4310653 25125383

[12] Lefaucheur C Viglietti D Bentlejewski C Duong van Huyen JP Vernerey D Aubert O. IgG Donor-Specific Anti-Human HLA Antibody Subclasses and Kidney Allograft Antibody-Mediated Injury. J Am Soc Nephrol (2016) 27:293–304. 10.1681/ASN.2014111120 26293822 PMC4696574

[13] Crespo M Yelamos J Redondo D Muntasell A Perez-Saez MJ Lopez-Montanes M. Circulating NK-Cell Subsets in Renal Allograft Recipients With Anti-HLA Donor-Specific Antibodies. Am J Transplant (2015) 15:806–14. 10.1111/ajt.13010 25656947

[14] Hidalgo LG Sis B Sellares J Campbell PM Mengel M Einecke G. NK Cell Transcripts and NK Cells in Kidney Biopsies From Patients With Donor-Specific Antibodies: Evidence for NK Cell Involvement in Antibody-Mediated Rejection. Am J Transplant (2010) 10:1812–22. 10.1111/j.1600-6143.2010.03201.x 20659089

[15] Gupta A Broin PO Bao Y Pullman J Kamal L Ajaimy M. Clinical and Molecular Significance of Microvascular Inflammation in Transplant Kidney Biopsies. Kidney Int (2016) 89:217–25. 10.1038/ki.2015.276 26422506

[16] Haas M Sis B Racusen LC Solez K Glotz D Colvin RB. Banff 2013 Meeting Report: Inclusion of C4d-Negative Antibody-Mediated Rejection and Antibody-Associated Arterial Lesions. Am J Transplant (2014) 14:272–83. 10.1111/ajt.12590 24472190

[17] Sablik KA Clahsen-van Groningen MC Looman CWN Damman J Roelen DL van Agteren M. Chronic-Active Antibody-Mediated Rejection With or Without Donor-Specific Antibodies has Similar Histomorphology and Clinical Outcome - A Retrospective Study. Transpl Int (2018) 31:900–8. 10.1111/tri.13154 29570868

[18] Senev A Coemans M Lerut E Van Sandt V Daniels L Kuypers D. Histological Picture of Antibody-Mediated Rejection Without Donor-Specific Anti-HLA Antibodies: Clinical Presentation and Implications for Outcome. Am J Transplant (2019) 19:763–80. 10.1111/ajt.15074 30107078

[19] Dragun D Müller DN Bräsen JH Fritsche L Nieminen-Kelhä M Dechend R. Angiotensin II Type 1-Receptor Activating Antibodies in Renal-Allograft Rejection. N Engl J Med (2005) 352(6):558–69. 10.1056/NEJMoa035717 15703421

[20] Reinsmoen NL Lai CH Heidecke H Haas M Cao K Ong G. Anti-Angiotensin Type 1 Receptor Antibodies Associated With Antibody Mediated Rejection in Donor HLA Antibody Negative Patients. Transplantation (2010) 90(12):1473–7. 10.1097/TP.0b013e3181fd97f1 21030904

[21] Opelz G. Non-HLA Transplantation Immunity Revealed by Lymphocytotoxic Antibodies. Lancet (2005) 365:1570–6. 10.1016/S0140-6736(05)66458-6 15866311

[22] Delville M Charreau B Rabant M Legendre C Anglicheau D. Pathogenesis of Non-HLA Antibodies in Solid Organ Transplantation: Where Do We Stand? Hum Immunol (2016) 77:1055–62. 10.1016/j.humimm.2016.05.021 27237040

[23] Delville M Lamarthee B Pagie S See SB Rabant M Burger C. Early Acute Microvascular Kidney Transplant Rejection in the Absence of Anti-HLA Antibodies Is Associated With Preformed IgG Antibodies Against Diverse Glomerular Endothelial Cell Antigen. J Am Soc Nephrol (2019) 30:692–709. 10.1681/ASN.2018080868 30850439 PMC6442343

[24] Philogene MC Bagnasco S Kraus ES Montgomery RA Dragun D Leffell MS. Anti-Angiotensin II Type 1 Receptor and Anti-Endothelial Cell Antibodies: A Cross-Sectional Analysis of Pathological Findings in Allograft Biopsie. Transplantation (2017) 101(3):608–15. 10.1097/TP.0000000000001231 PMC5319389 27222934

[25] Zitzner JR Shah S Jie C Wegner W Tambur AR Friedewald JJ. A Prospective Study Evaluating the Role of Donor-Specific Anti-Endothelial Crossmatch (XM-ONE Assay) in Predicting Living Donor Kidney Transplant Outcome. Hum Immunol (2013) 74:1431–6. 10.1016/j.humimm.2013.06.007 23777928

[26] Gareau AJ Wiebe C Pochinco D Gibson IW Ho J Rush DN. Pre-Transplant AT1R Antibodies Correlate With Early Allograft Rejection. Transpl Immunol (2018) 46:29–35. 10.1016/j.trim.2017.12.001 29217423

[27] Lim WH Wong G Heidt S Claas FHJ. Novel Aspects of Epitope Matching and Practical Application in Kidney Transplantation. Kidney Int (2018) 93(2):314–24. 10.1016/j.kint.2017.08.008 29061333

[28] Duquesnoy RJ Kamoun M Baxter-Lowe LA Woodle ES Bray RA Claas FH. Should HLA Mismatch Acceptability for Sensitized Transplant Candidates be Determined at the High-Resolution Rather Than the Antigen Level? Am J Transplant (2015) 15(4):923–30. 10.1111/ajt.13167 25778447

[29] Wiebe C Pochinco D Blydt-Hansen TD Ho J Birk PE Karpinski M. Class II HLA Epitope Matching - A Strategy to Minimize De Novo Donor-Specific Antibody Development and Improve Outcomes. Am J Transplant (2013) 13:3114–22. 10.1111/ajt.12478 24164958

[30] Wiebe C Rush DN Nevins TE Birk PE Blydt-Hansen T Gibson IW. Class II Eplet Mismatch Modulates Tacrolimus Trough Levels Required to Prevent Donor-Specific Antibody Developmen. J Am Soc Nephrol (2017) 28:3353–62. 10.1681/ASN.2017030287 PMC5661295 28729289

[31] Tafulo S Malheiro J Santos S Dias L Almeida M Martins S. Degree of HLA Class II Eplet Mismatch Load Improves Prediction of Antibody-Mediated Rejection in Living Donor Kidney Transplantation. Hum Immunol (2019) 80(12):966–75. 10.1016/j.humimm.2019.09.010 31604581

[32] Besarani D Cerundolo L Smith JD Procter J Barnardo MC Roberts IS. Role of Anti-Vimentin Antibodies in Renal Transplantation. Transplantation (2014) 98(1):72–8. 10.1097/01.TP.0000443224.66960.37 24978037

[33] Gimeno J Redondo D Perez-Saez MJ Naranjo-Hans D Pascual J Crespo M. Impact of the Banff 2013 Classification on the Diagnosis of Suspicious Versus Conclusive Late Antibody-Mediated Rejection in Allografts Without Acute Dysfunction. Nephrol Dial Transplant (2016) 31:1938–46. 10.1093/ndt/gfw223 27312147

[34] Redondo-Pachon D Perez-Saez MJ Mir M Gimeno J Llinas L Garcia C. Impact of Persistent and Cleared Preformed HLA DSA on Kidney Transplant Outcomes. Hum Immunol (2018) 79:424–31. 10.1016/j.humimm.2018.02.014 29524568

[35] Reinsmoen NL Mirocha J Ensor CR Marrari M Chaux G Levine DJ. A 3-Center Study Reveals New Insights Into the Impact of Non-HLA Antibodies on Lung Transplantation Outcom. Transplantation (2017) 101(6):1215–21. 10.1097/TP.0000000000001389 27973391

[36] Zhang Q Cecka JM Gjertson DW Ge P Rose ML Patel JK. HLA and MICA: Targets of Antibody-Mediated Rejection in Heart Transplantation. Transplantation (2011) 91(10):1153–8. 10.1097/TP.0b013e3182157d60 PMC3563270 21544036

[37] Duquesnoy RJ. A Structurally Based Approach to Determine HLA Compatibility at the Humoral Immune Level. Hum Immunol (2006) 67:847–62. 10.1016/j.humimm.2006.08.001 PMC2169290 17145365

[38] Luque S Lucia M Melilli E Lefaucheur C Crespo M Loupy A. Value of Monitoring Circulating Donor-Reactive Memory B Cells to Characterize Antibody-Mediated Rejection After Kidney Transplantation. Am J Transplant (2019) 19:368–80. 10.1111/ajt.15055 30085394

[39] Zhang Q Reed EF. The Importance of Non-HLA Antibodies in Transplantation. Nat Rev Nephrol (2016) 12:484–95. 10.1038/nrneph.2016.88 PMC5669045 27345243

[40] Sanchez-Zapardiel E Castro-Panete MJ Mancebo E Morales P Laguna-Goya R Morales JM. Early Renal Graft Function Deterioration in Recipients With Preformed Anti-MICA Antibodies: Partial Contribution of Complement-Dependent Cytotoxicity. Nephrol Dial Transplant (2016) 31:150–60. 10.1093/ndt/gfv308 26323481

[41] Le Bas-Bernardet S Hourmant M Coupel S Bignon JD Soulillou JP Charreau B. Non-HLA-Type Endothelial Cell Reactive Alloantibodies in Pre-Transplant Sera of Kidney Recipients Trigger Apoptosis. Am J Transplant (2003) 3:167–77. 10.1034/j.1600-6143.2003.00021.x 12603212

[42] Butler CL Hickey MJ Jiang N Zheng Y Gjertson D Zhang Q. Discovery of Non-HLA Antibodies Associated With Cardiac Allograft Rejection and Development and Validation of a Non-HLA Antigen Multiplex Panel: From Bench to Bedside. Am J Transplant (2020) 20(10):2768–80. 10.1111/ajt.15863 PMC7494540 32185871

[43] Porcheray F DeVito J Yeap BY Xue L Dargon I Paine R. Chronic Humoral Rejection of Human Kidney Allografts Associates With Broad Autoantibody Responses. Transplantation (2010) 89(10):1239–46. 10.1097/TP.0b013e3181d72091 PMC3864120 20445487

[44] Jackson AM Sigdel TK Delville M Hsieh SC Dai H Bagnasco S. Endothelial Cell Antibodies Associated With Novel Targets and Increased Rejection. J Am Soc Nephrol (2015) 26:1161–71. 10.1681/ASN.2013121277 PMC4413753 25381426

[45] Dinavahi R George A Tretin A Akalin E Ames S Bromberg JS. Antibodies Reactive to Non-HLA Antigens in Transplant Glomerulopathy. J Am Soc Nephrol (2011) 22:1168–78. 10.1681/ASN.2010111183 PMC3103737 21566057

[46] Lefaucheur C Viglietti D Bouatou Y Philippe A Pievani D Aubert O. Non-HLA Agonistic Anti-Angiotensin II Type 1 Receptor Antibodies Induce a Distinctive Phenotype of Antibody-Mediated Rejection in Kidney Transplant Recipients. Kidney Int (2019) 96(1):189–201. 10.1016/j.kint.2019.01.030 31005275

[47] Zhang J Wang M Liang J Zhang M Liu XH Ma L. The Presence of Anti-Angiotensin II Type-1 Receptor Antibodies Adversely Affect Kidney Graft Outcome. Int J Environ Res Public Health (2017) 14:500. 10.3390/ijerph14050500 PMC5451951 28486415

[48] Giral M Foucher Y Dufay A Duong Van Huyen JP Renaudin K Moreau A. Pretransplant Sensitization Against Angiotensin II Type 1 Receptor Is a Risk Factor for Acute Rejection and Graft Loss. Am J Transplant (2013) 13:2567–76. 10.1111/ajt.12397 23919486

[49] Taniguchi M Rebellato LM Cai J Hopfield J Briley KP Haisch CE. Higher Risk of Kidney Graft Failure in the Presence of Anti-Angiotensin II Type-1 Receptor Antibodies. Am J Transplant (2013) 13:2577–89. 10.1111/ajt.12395 23941128

[50] Einecke G Sis B Reeve J Mengel M Campbell PM Hidalgo LG. Antibody-Mediated Microcirculation Injury Is the Major Cause of Late Kidney Transplant Failure. Am J Transplant (2009) 9:2520–31. 10.1111/j.1600-6143.2009.02799.x 19843030

[51] Sellares J de Freitas DG Mengel M Reeve J Einecke G Sis B. Understanding the Causes of Kidney Transplant Failure: The Dominant Role of Antibody-Mediated Rejection and Nonadherence. Am J Transplant (2012) 12:388–99. 10.1111/j.1600-6143.2011.03840.x 22081892

[52] Arias-Cabrales C Redondo-Pachón D Pérez-Sáez MJ Gimeno J Sánchez-Güerri I Bermejo S. Renal Graft Survival According to Banff 2013 Classification in Indication Biopsies. Nefrología (English Ed) (2016) 36(6):660–6. 10.1016/j.nefroe.2016.05.012 27595515

[53] Koenig A Chen CC Marcais A Barba T Mathias V Sicard A. Missing Self Triggers NK Cell-Mediated Chronic Vascular Rejection of Solid Organ Transplants. Nat Commun (2019) 10(1):5350. 10.1038/s41467-019-13113-5 31767837 PMC6877588

[54] Senev A Emonds MP Van Sandt V Lerut E Coemans M Sprangers B. Clinical Importance of Extended Second Field High-Resolution HLA Genotyping for Kidney Transplantation. Am J Transplant (2020) 20:3367–78. 10.1111/ajt.15938 PMC7754319 32337773

