# Supplementary material for: Non-HLA Antibodies and Epitope Mismatches in Kidney Transplant Recipients With Histological Antibody-Mediated Rejection
Source: Front Immunol. 2021 Jul 6;12:703457. doi: 10.3389/fimmu.2021.703457 (PMC8300190; doi:10.3389/fimmu.2021.703457)
Supplement: Supplementary file 1 [file DataSheet_1.docx]

Supplementary Material

# Supplementary Figures and Tables

## Supplementary Figures


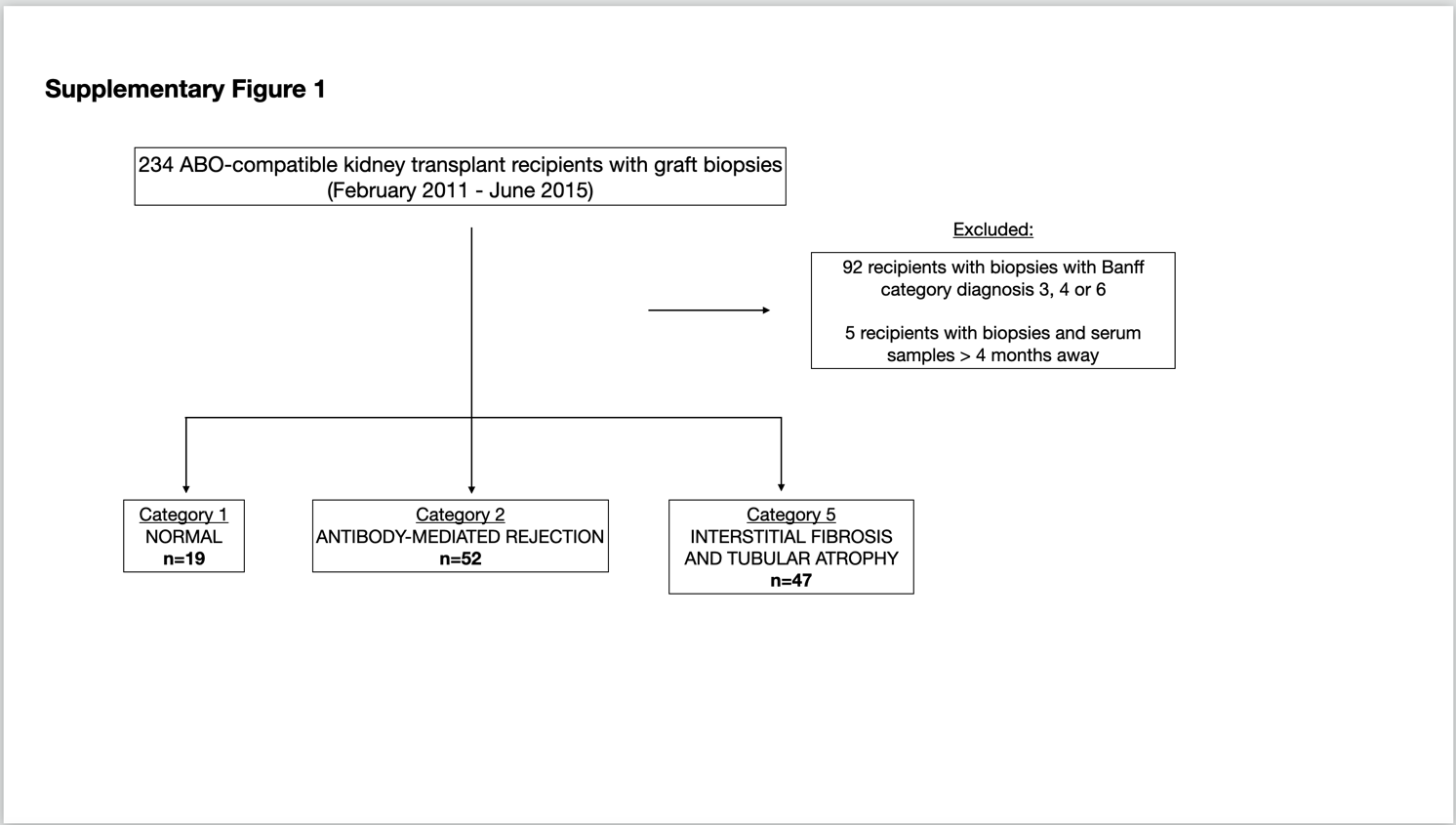


**Supplementary Figure 1.** Patient flow-chart.

**
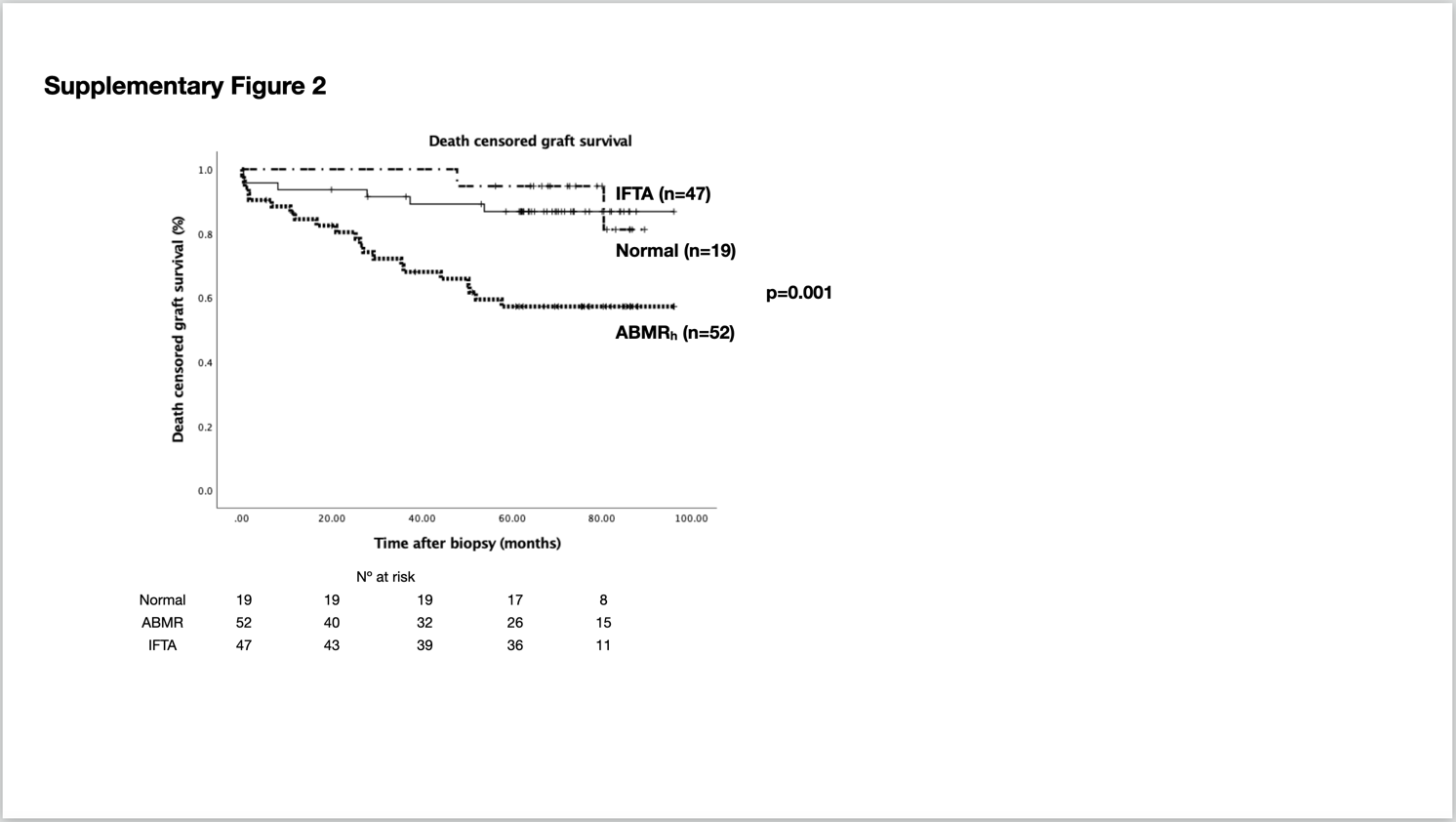
**

**Supplementary Figure 2**. Death censored graft survival in patients with normal, ABMR_h_ and IFTA biopsies. Kaplan-Meier survival curves representing death censored graft survival. ABMR_h_: antibody-mediated rejection histology; IFTA: interstitial fibrosis and tubular atrophy.

## Supplementary Tables

**Supplementary Table 1**.

(**A**) Comparison of post-transplant non-HLA antibodies between ABMR_h_DSA_pos_ and ABMR_h_DSA_neg_ cases.

|  | **ABMR_h_DSA_pos_ (n=38)** | **ABMR_h_DSA_neg_ (n=14)** | **p-value** |
| --- | --- | --- | --- |
| Post-transplant AT_1_R-Ab (yes, %) | 10 (26.3%) | 2 (14.3%) | 0.48 |
| Post-transplant ETAR-Ab (yes, %) | 6 (15.8%) | 1 (7.1%) | 0.66 |
| Post-transplant MICA-Ab (yes, %) | 6 (15.8%) | 2 (14.3%) | 1.00 |
| Post-transplant EC-XM (positive, %) ^$^ | 4 (13.3%) | 0 (0%) | 0.30 |

^$^ From 30 ABMR_h_DSA_pos_ and 13 ABMR_h_DSA_neg_ cases.

ABMR_h_: antibody-mediated rejection histology; AT_1_R-Ab: antibodies against angiotensin II type 1 receptor; EC-XM: crossmatch with primary aortic endothelial cells; ETAR-Ab: antibodies against endothelin-1 type A receptor; MICA-Ab: antibodies against major histocompatibility complex class I related chain A.

(**B**) Comparison of post-transplant HLA and non-HLA antibodies between ABMR_h_DSA_pos_ and non-ABMR_h_DSA_pos_ cases (normal histology, IFTA and ABMR_h_DSA_neg_ cases).

|  | **ABMR_h_DSA_pos_ (n=38)** | **No ABMR_h_DSA_pos_ (n=80)** | **p-value** |
| --- | --- | --- | --- |
| Post-transplant HLA-DSA (yes, %) | 38 (100%) | 11 (13.8%) | **<0.001** |
| Post-transplant AT_1_R-Ab (yes, %) | 10 (26.3%) | 20 (25%) | 0.88 |
| Post-transplant ETAR-Ab (yes, %) | 6 (15.8%) | 13 (16.3%) | 0.95 |
| Post-transplant MICA-Ab (yes, %) | 6 (15.8%) | 9 (11.3%) | 0.56 |
| Post-transplant EC-XM (positive, %) ^#^ | 4 (13.3%) | 4 (5.5%) | 0.23 |

^#^30 ABMR_h_DSA_pos_ and 73 non-ABMR_h_DSA_pos_ cases.

AT_1_R-Ab: antibodies against angiotensin II type 1 receptor; EC-XM: crossmatch with primary aortic endothelial cells; ETAR-Ab: antibodies against endothelin-1 type A receptor; HLA-DSA: HLA donor-specific antibodies; MICA-Ab: antibodies against major histocompatibility complex class I related chain A.
